# Prediction of severe thunderstorm events with ensemble deep learning and radar data

**DOI:** 10.1038/s41598-022-23306-6

**Published:** 2022-11-21

**Authors:** Sabrina Guastavino, Michele Piana, Marco Tizzi, Federico Cassola, Antonio Iengo, Davide Sacchetti, Enrico Solazzo, Federico Benvenuto

**Affiliations:** 1 The MIDA Group, Dipartimento di Matematica, Università di Genova, Genova, Italy; 2 CNR - SPIN Genova, Genova, Italy; 3 ARPAL, Genova, Italy; 4 ARPA Lombardia, Milan, Italy

**Keywords:** Atmospheric science, Natural hazards, Applied mathematics, Scientific data, Software

## Abstract

The problem of nowcasting extreme weather events can be addressed by applying either numerical methods for the solution of dynamic model equations or data-driven artificial intelligence algorithms. Within this latter framework, the most used techniques rely on video prediction deep learning methods which take in input time series of radar reflectivity images to predict the next future sequence of reflectivity images, from which the predicted rainfall quantities are extrapolated. Differently from the previous works, the present paper proposes a deep learning method, exploiting videos of radar reflectivity frames as input and lightning data to realize a warning machine able to sound timely alarms of possible severe thunderstorm events. The problem is recast in a classification one in which the extreme events to be predicted are characterized by a an high level of precipitation and lightning density. From a technical viewpoint, the computational core of this approach is an ensemble learning method based on the recently introduced value-weighted skill scores for both transforming the probabilistic outcomes of the neural network into binary predictions and assessing the forecasting performance. Such value-weighted skill scores are particularly suitable for binary predictions performed over time since they take into account the time evolution of events and predictions paying attention to the value of the prediction for the forecaster. The result of this study is a warning machine validated against weather radar data recorded in the Liguria region, in Italy.

## Introduction

One of the most interesting problems in weather forecasting is the prediction of extreme rainfall events such as severe thunderstorms possibly leading to flash floods. This problem is very challenging especially when we consider areas characterized by a complex, steep orography close to a coastline, where intense precipitation can be enhanced by specific topographic features: this is the case for the example of Liguria, an Italian region located on the northwest Mediterranean Sea and characterized by the presence of mountains over 2000 m high at only a few kilometres away from the coastline. This specific morphology gives rise to several catchments with steep slopes and limited extension^1^. Autumn events, when deep Atlantic troughs more easily enter the Mediterranean area and activate very moist and unstable flow lifted by the mountain range, may cause catastrophic flooding on these coastal areas, which are characterized by a high population density (see^2,3^ for a review of the climatology and typical atmospheric configurations of extreme precipitation over the Mediterranean area). Just as an example, the November 4th 2011 flood in Genoa caused six deaths and economic damage up to 100 million euros^4–7^). A common feature in these extreme events is the presence of a quasi-stationary convective system with a spatial extension of few kilometers^8–12^

Medium and long range either deterministic or ensemble Numerical Weather Prediction (NWP) models still struggle to correctly predict both the intensity and the location of these events, which can be triggered and enhanced by very small-scale features. High resolution convection-permitting NWP models manage to partly return a more realistic description of the dynamics of severe thunderstorms. Many studies addressed the role played by different components or settings of NWP models in order to better describe severe convective systems over the Liguria area, such as model resolution, initial conditions, microphysics schemes or small-scale patterns of the sea surface temperature^6,13–19^.

However, the intrinsically limited predictability of convective systems requires the use of shorter-term *nowcasting* models, e.g. in order to feed automatic early warning systems, which may support meteorologists and hydrologists in providing accurate and reliable forecasts and thus reducing the consequences of these extreme events. These forecasting systems typically rely on two kinds of approaches. On the one hand, either stochastic or deterministic models are formulated utilizing partial differential equations in fluid dynamics, and numerical methods are implemented for their reduction, nesting hydrological models into meteorological ones^20–22^. On the other hand, more recent data-driven techniques take as input a time series of radar (and in case satellite) images belonging to a historical archive and provide as output a synthetic image representing the prediction of the radar signal at a subsequent time point; this approach can rely on some extrapolation technique, e.g. based on a storm-tracking system^23^ or a diffusive process in Fourier space^24^, or on deep learning networks^25–36^. Mixed techniques have been also proposed, blending NWP outputs with data-driven synthetic predictions^37^. The aim of these studies is to make time series prediction by exploiting image-based deep learning techniques, such as U-net^26^, Convolutional Long Short-Term Memory (ConvLSTM)^28^, improvements of ConvLSTM as Trajectory Gated Recurrent Unit (TrajGRU)^30,33,34^, and Generative Adversial Networks (GANs)^35,36^, which produce reflectivity images in the next future. From the predicted reflectivity images the rainfall quantity can be extrapolated but no indication of the presence of lightning can be provided. In our work, we focus on the forecasting of extreme thunderstorm events and therefore previous methods mentioned above do not directly apply to our problem. On the contrary, we present a novel method which recasts the problem into a classification one by using the lightning density as a fundamental feature for characterizing an extreme event. Towards this aim, we exploit a deep neural network, originally conceived for video classification, to predict the probability that an extreme event occurs. We use as input time series of multichannel radar images and we define the labels on the basis of a certain level of precipitation and lightning density. The deep-learning model combines a convolutional neural network (CNN) with a long short-term memory (LSTM) network^38,39^ in order to construct a long-term recurrent convolutional network (LRCN)^40^. The prediction assessment is performed by means of the recently introduced value-weighted skill scores^41^ which allows ranking prediction errors on the basis of their distribution along time, preferring to show up a warning well in advance of the actual occurrence of an event rather than not to show it at all. Finally, we exploit the iterative nature of the network training process to collect a set of predictions from which we select a subset of valuable ones on the basis of their value-weighted skill score. This procedure falls within the class of ensemble learning techniques. We remark that the term “ensemble” as used here refers to deep learning methods and not to the NWP algorithms. The main methodological novelties of this approach are the following.

1. The prediction problem is reformulated into a binary classification one in which labels depend on both heavy rainfall conditions and lightning density;
2. forecasting verification is performed by the use of value-weighted skill scores on the basis of an automatic ensemble strategy.

Other works have been translated the forecasting problem into a binary prediction , but the focus was on moderate rain, i.e. when the rainfall is beyond a certain threshold, mainly $>5$ mm/h or at most $>30$ mm/h. To our knowledge, the present work is the first attempt to predict severe thunderstorm events on the basis of lightnings and radar video data. Moreover, forecast verification is completely different with respect to previous works. Usually, skill scores compare the predictions with observations in a time independent way, i.e, a score remains unchanged if we permute the temporal order of events and predictions in the same way. On the contrary, the value-weighted skill scores take into account the time evolution of events and predictions paying attention on the value of the prediction for the forecaster. Indeed, this approach provides probabilistic outcomes concerning the event occurrence and related quantitative parameters, thus realizing an actual warning machine for the forecasting of extreme events. The results of this study is a data-driven warning system for supporting the decision making in the case of extreme rainfall events tailored for the Ligurian region. This system takes advantage of the value-weighted skill scores which, in the framework of an ensemble learning approach, allow the deep network to provide predictions more accurate than those obtained when standard quality-based skill scores are applied.

The paper is organized as follows. In “Constant altitude plan position indicator reflectivity data in Liguria” section we describe the considered weather radar and lightning data, and in “Long-term recurrent convolutional network” section we give details on the architecture of the LRCN model used in this study. In “Ensemble deep learning” section we recall the definition of value-weighted skill scores, and we describe the proposed ensemble deep learning technique. In “Experimental results” section we show the effectiveness of the method in prediction of extreme rainfall events using radar-based data. Our conclusions are offered in “Conclusions and future work” section.

## Constant altitude plan position indicator reflectivity data in Liguria

Precipitation activity and locations of rain, showers, and thunderstorms are commonly monitored in real-time by polarimetric Doppler weather radars; return echoes from targets (such as hydrometeors) allow the measurement of the reflectivity field on different conical surfaces, one at each elevation angle of the radar; however, reflectivity values at a certain height can be interpolated to 2D maps, which are also known as Constant Altitude Plan Position Indicator (CAPPI) images^42^; such a representation is particularly useful for compositing reflectivity data measured by different radars over overlapping regions, returning a reflectivity field for the larger area covered by a radar network.

**Figure 1:**
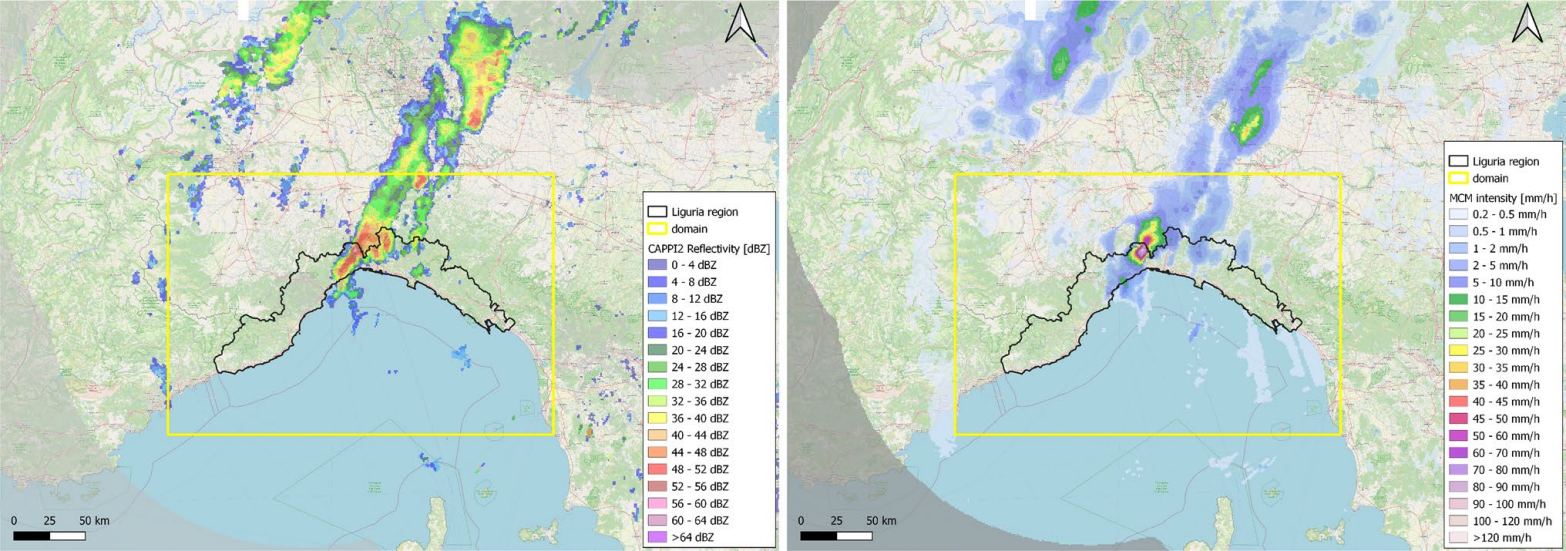
An example of a 2-km CAPPI reflectivity frame (left) and an MCM rain rate frame (right) (both referred to 21/10/2019 23:00 UTC); the selected area surrounding Liguria is delimited in yellow. The maps are downloaded from OpenStreetMap.

In our study CAPPI reflectivity fields measured by the Italian Radar Network within the Civil Protection Department are considered. CAPPI images, measured in dBZ, are sampled every 10 minutes at a spatial resolution of $0.005^{\circ } \simeq 0.56$ km in latitude and $0.005^{\circ } \simeq 0.38$ km in longitude. We used CAPPI images at three different heights (2 km, 3 km, and 5 km above sea level (ASL)) and cut each image over an area comprising the Liguria region (as shown in Fig. 1). In detail, for each image the latitude ranges in [$43.4^{\circ }$ N, $45.0^{\circ }$ N] and the longitude ranges in [$7.1^{\circ }$ E, $10.4^{\circ }$ E], so that images have size $321 \times 661$ and cover an area of about 180 km in latitude and 250 km in longitude. We used 1.5-hour-long movies of CAPPI images to construct temporal feature sequences to predict the occurrence of extreme rainfall event in the hour after the last time step.

The training set exploited to optimize the LRCN is generated by means of a labeling procedure involving modified conditional merging (MCM) data and lightning data. MCM data^43^ combine radar rain estimates and rain gauge measurements with an hourly frequency and provide the amount of rainfall integrated over 1 hour (in these data the content of each pixel is measured in mm per hour and the spatial resolution is $0.013267^{\circ } \simeq 1$ km in longitude and $0.008929^{\circ } \simeq 1$ km in latitude; see Fig. 1). Lightning data are recorded by the LAMPINET network of Military Aeronautics^44^ and have a resolution of 1 microsecond.

The labeling process associates each CAPPI video to the concept of severe convective rainfall event, whose definition relies on the following two items:

- MCM data must contain at least 3 contiguous pixels exceeding 50 mm/h within the selected area;
- at least 10 lightning strikes must occur in a 10-minute time range in the area comprising 5 km around each one of the MCM pixels with over-threshold content.

It is worth noticing that 50 mm/h is regarded as a threshold for heavy rain in the Liguria region; however, the first condition accounts for the fact that an over-threshold value associated to an isolated pixel may be associated to spurious non-meteorological echoes like, for instance, the passage of a plane. On the other hand, the second condition implies that the extreme events considered must always involve the occurrence of thunderstorms.

## Long-term recurrent convolutional network

**Figure 2:**
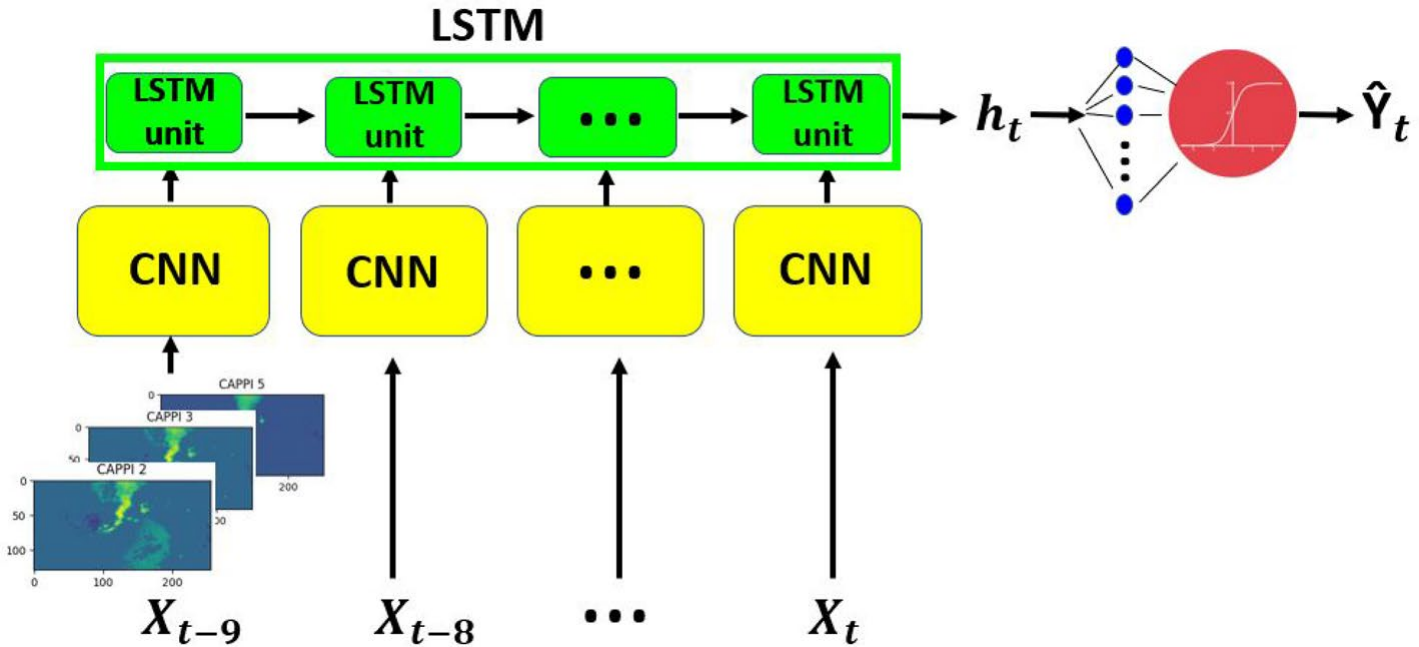
The LRCN architecture.

The idea of this work is to address the prediction of extreme events in the short term as a radar image video classification problem. Following the work of^40^ we propose the use of a Long-term recurrent convolutional network (LRCN) which combines a convolutional neural network (CNN) and a long short-term memory (LSTM) network to create spatio-temporal deep learning models^45,46^. In this application, the input is made of time series of 10 radar reflectivity images (representing a video 1.5 hours long) at the three CAPPI 2, CAPPI 3 and CAPPI 5 levels, which refer to 2 km, 3 km and 5 km ASL, respectively. Images have been resized to a $128 \times 256$ pixel size in order to guarantee a good trade-off between computational efficiency and image resolution. The CNN is used to automatically extract spatial features from the image set. The features are decomposed into sequential components and fed to the LSTM network to be analyzed. Finally, the output of the LSTM layer is fed into the fully connected layer, and the sigmoid activation function is applied to generate the probability distribution of the positive class. Figure 2 shows the architecture of the LRCN model.

**Figure 3:**
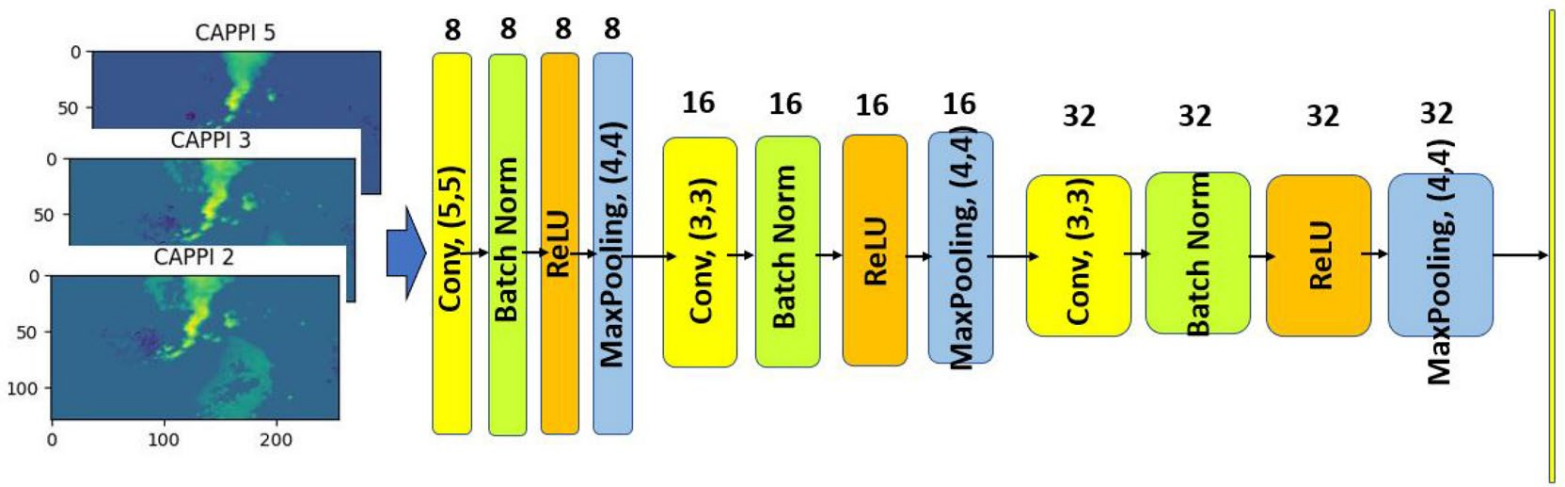
The CNN architecture.

In this work the CNN architecture of the LRCN model consists in three blocks, each one composed by a convolutional layer with stride (2, 2), followed by a batch normalization layer to improve stability; the Rectified Linear Unit (ReLU) function^47^ is adopted as an activation function and the max pooling operation with size (4,4) and stride (2, 2) is applied. We initialize all the convolutional weights by sampling from the scaled uniform distribution^48^. The three convolutional layers are characterized by 8, 16 and 32 kernels with size (5, 5), (3, 3) and (3, 3), respectively. The input are sequences of size (*T*, 128, 256, 3), where *T* represents the number of frames in each movie, 128 and 256 correspond to the image size (in pixel) and 3 represents the three levels of CAPPI data. In all operations we take advantage of the “Timedistributed” layer, available in the Keras library^49^, which allows the in parallel training of the *T* convolutional flows. Figure 3 illustrates this CNN architecture. Then, the CNN output is flattened to create the sequence of feature vectors to feed into the LSTM network. In our experiments, the LSTM layer has 50 hidden neurons. Finally, the dropout layer is used to prevent overfitting^50^: the dropout value is set to 0.5, meaning that $50\%$ of neurons are randomly dropped from the neural network during training in each iteration. The hyperparameters of the NN are estimated by an empirical trial-and-error optimization process on several experiments.

### Loss function

Once the architecture of the NN is set up, we can denote with $\theta$ the NN weights and we can interpret the NN as a map $f_{\theta }$, mapping a sample *X* to a probability outcome $f_{\theta }(X)\in [0,1]$, since the sigmoid activation function is applied in the last layer. We recall that, in our application, the sample *X* is a video of CAPPI reflectivity images and $f_{\theta }(X)$ represents the predicted probability of the occurrence of an extreme rainfall event in the next hour after the end time of the CAPPI video *X* within the selected area (in fact, we are not interested in the exact location of the possible event). In the training process we consider an optimization problem

1 $$\begin{aligned} \min _{\mathbf {\theta }} \ell (F_{\mathbf {\theta }}(\textbf{X}),\textbf{Y}), \end{aligned}$$

where $\{\textbf{X},\textbf{Y}\}=\{(X_i,Y_i)\}_{i=1}^n$ is the training set ($Y_i$ represents the actual label of the sample $X_i$ according to the definition given in “Constant altitude plan position indicator reflectivity data in Liguria”) Section, $F_{\mathbf {\theta }}(\textbf{X})=(f_{\mathbf {\theta }}(X_i))_i$ represents the probability outcomes of the NN on the set $\textbf{X}$ and $\ell$ represents the loss function measuring the discrepancy between the true label $\textbf{Y}$ and the predicted output $F_{\theta }(\textbf{X})$. In classification problems the most used loss function is the binary cross-entropy. In the case of imbalanced data sets, modifications of the cross-entropy loss are considered, such as the following one:

2 $$\begin{aligned} \ell (F_{\mathbf {\theta }}(\textbf{X}),\textbf{Y})=-\left( \sum _{i=1}^n \beta _1 Y_i\log (f_{\mathbf {\theta }}(X_i)) + \beta _0 (1-Y_i)\log (1-f_{\mathbf {\theta }}(X_i)) \right) , \end{aligned}$$

where $\beta _0,\beta _1$ are positive weights defined according to the data set imbalance. We define the weights as

3 $$\begin{aligned} \beta _1=\frac{1}{\#\{i\in \{1,\dots ,n\} : Y_i=1\}} \text { and } \beta _0=\frac{1}{\#\{i\in \{1,\dots ,n\} : Y_i=0\}}, \end{aligned}$$

where $Y_i=1$ indicates the presence of extreme rainfall event and $Y_i=0$ indicates the absence of extreme rainfall event. We refer to the chosen loss function as the class balanced cross-entropy.

## Ensemble deep learning

During the iterative optimization process a set of deep neural networks $X \rightarrow f_\theta (X)$ by varying of $\theta$ is generated. The proposed ensemble deep learning technique selects a subset of this set as follows. For each $\theta$, it transforms the probabilistic outcome $f_\theta (X)$ of $f_\theta$ into a binary prediction and then it evaluates on the validation set such a prediction according to its value-weighted skill score. To describe this strategy in detail we start by the value-weighted skill score..

### Evaluation skill scores

The result of a binary classifier is usually evaluated by computing the confusion matrix, also known as the contingency table. Let us denote with ${\mathbb {M}}_{2,2}({\mathbb {N}})$ the set of 2-dimensional matrices with natural elements. Let $\textbf{Y}=(Y_i)\in \{0,1\}^n$ be a binary sequence representing the actual labels of a given dataset of examples, and let $\hat{\textbf{Y}}=(\hat{Y}_i)\in \{0,1\}^n$ be a binary sequence representing the prediction. Then the classical (quality-based) confusion matrix $\tilde{\textbf{C}}\in {\mathbb {M}}_{2,2}({\mathbb {N}})$ is given by:

$$\begin{aligned} \tilde{\textbf{C}}(\hat{\textbf{Y}},\textbf{Y}) = \begin{pmatrix} \text{TN} &{} \text{FP} \\ \text{FN} &{} \text{TP} \end{pmatrix}, \end{aligned}$$

where $\text{TP}=\sum _{i=1}^n \mathbbm {1}_{\{Y_i=1,\hat{Y}_i=1\}}$ represents the true positives, i.e. the number of samples correctly classified as the positive class; $\text{TN}=\sum _{i=1}^n \mathbbm {1}_{\{Y_i=0,\hat{Y}_i=0\}}$ represents the true negatives, i.e. the number of samples correctly classified as the negative class; $\text{FP}=\sum _{i=1}^n \mathbbm {1}_{\{Y_i=0,\hat{Y}_i=1\}}$ represents the false positives, i.e. the number of negative samples incorrectly classified as the positive class; $\text{FN}=\sum _{i=1}^n \mathbbm {1}_{\{Y_i=1,\hat{Y}_i=0\}}$ represents the false negatives, i.e. the number of positive samples incorrectly classified as the negative class.

A specific classical (quality-based) skill score is given by a map $\text{S}:{\mathbb {M}}_{2,2}({\mathbb {N}})\rightarrow {\mathbb {R}}$ defined on the confusion matrix $\tilde{\textbf{C}}$. In this study we considered two skill-scores, i.e., the critical success index (CSI)

4 $$\begin{aligned} \text{CSI}(\tilde{\textbf{C}}(\hat{\textbf{Y}},\textbf{Y}))=\frac{\text{TP}}{\text{TP}+\text{FP}+\text{FN}}, \end{aligned}$$

which is commonly used in meteorological applications^34^; and the true skill statistic (TSS)

5 $$\begin{aligned} \text{TSS}(\tilde{\textbf{C}}(\hat{\textbf{Y}},\textbf{Y}))=\frac{\text{TP}}{\text{TP} +\text{FN}}-\frac{\text{FP}}{\text{FP}+\text{TN}}~, \end{aligned}$$

which is particularly appropriate for imbalanced data sets^51^. The CSI varies from [0, 1], while the TSS varies from $[-1,1]$ and for both scores the optimal value is 1.

However, such metrics do not account for the distribution of predictions along time and are not able to provide a quantitative preference to those alarms that predict an event well in advance with respect to its actual occurrence, and to penalize predictions sounding delayed false alarms. To overcome these limitations, value-weighted confusion matrices have been introduced^41^. The aim of the value-weighted approach is to mitigate errors such as false positives that precede false negatives (the case of predictions well in advance) and false negatives which are preceded by true positives (the case of on going events already predicted) as they have little impact on the prediction from the point of view of the forecaster. In fact, a value-weighted confusion matrix is defined as

6 $$\begin{aligned} \textbf{C}_{\text{w}}(\hat{\textbf{Y}},\textbf{Y}) = \begin{pmatrix} \text{TN} &{} \text{wFP} \\ \text{wFN} &{} \text{TP} \end{pmatrix}, \end{aligned}$$

with

7 $$\begin{aligned} \text{wFP}= \sum _{i=1}^n w(z^-_i,z^+_i)\mathbbm {1}_{\{Y_i=0,\hat{Y}_i=1\}}, \end{aligned}$$

8 $$\begin{aligned} \text{wFN}= \sum _{i=1}^n w({\hat{z}}^+_i,{\hat{z}}^-_i) \mathbbm {1}_{\{Y_i=1,\hat{Y}_i=0\}} ~. \end{aligned}$$

where the weights $w(z^-_i,z^+_i)$ and $w(z^-_i,z^+_i)$ are constructed as follows. First, the function *w* is

9 $$\begin{aligned} w(s,t)= {\left\{ \begin{array}{ll} 2 &{}\quad \text {if}~ s,t\equiv 0 \\ 1-\max (\mathrm w \circ t) &{}\quad \text{ otherwise } \end{array}\right. } \end{aligned}$$

where $\text{w}:=\left( \frac{1}{2},\frac{1}{3}, \ldots , \frac{1}{T+1} \right)$ and $\mathrm w \circ t$ indicates the element-wise product. Second, given the label $Y_i$ observed at the sampled time *i*, then $z^-_i = (Y_{i-1},Y_{i-2},\ldots ,Y_{i-T})$, is the sequence of the *T* elements before $Y_i$ and $z^+_i = (Y_{i+1},Y_{i+2},\ldots ,Y_{i+T})$ is the sequence of the *T* elements after $Y_i$. Analogously, given the label ${\hat{Y}}_i$ predicted at time *i*, then ${\hat{z}}^-_i = ({\hat{Y}}_{i-1},{\hat{Y}}_{i-2},\ldots ,{\hat{Y}}_{i-T})$, and ${\hat{z}}^+_i = ({\hat{Y}}_{i+1},{\hat{Y}}_{i+2},\ldots ,{\hat{Y}}_{i+T})$. The weight function $w:{\mathbb {R}}^T \times {\mathbb {R}}^T \rightarrow {\mathbb {R}}$ is then constructed in such a way to emphasize false positives associated with alarms predicted in the middle of $2T+1$-long time windows when no actual event occurs and false negatives associated with missed events in the middle of $2T+1$-long time windows in which no alarm is raised.

The introduction of this value-weighted confusion matrix allows the construction of the associated value-weighted Critical Success Index wCSI and the value-weighted True Skill Statistic wTSS, respectively.

### Ensemble strategy

We consider an ensemble procedure to provide an automatic classifier from the probability outcomes provided by the deep NN. Consider the first *N* epochs of the training process of the deep neural network $f_{\mathbf {\theta }}$. Denote with $\theta _j :=\theta _j(\{\textbf{X},\textbf{Y}\})$ the neural netwrork weights for each epoch *j* computed from the training set. The procedure has been introduced in^41^, and it can be summarized in the following steps:

1. For each epoch *j* we select the classification threshold $\overline{\tau }_j$, i.e. the real number that maximizes a given skill score
  10 $$\begin{aligned} \overline{\tau }_j = \arg \max _{\tau \in [0,1]} \text{S}(\textbf{C}(P_{\theta _j}^{\tau }(\textbf{X}),\textbf{Y}))). \end{aligned}$$
  where $P_{\theta _j}^{\tau }(\textbf{X}) :=(\textbf{1}_{\{f_{\theta _j}(X_i) >\tau \}})_{i=1,\ldots ,n}$ is the binary prediction on the set of samples $\textbf{X}$ and $\textbf{1}_{\{\cdot \}}$ denotes the indicator function. Then, we denote by
  11 $$\begin{aligned} \overline{P}_{\theta _j}(\textbf{X}) :=P_{\theta _j}^{ \overline{\tau } }(\textbf{X}) \end{aligned}$$
  the binary prediction on the set $\textbf{X}$ obtained by using the optimized threshold value.
2. Choose the subset of valuable predictions by selecting the predictors with a skill score higher than a given a quality level $\alpha$ on the validation set $\{\tilde{\textbf{X}},\tilde{\textbf{Y}}\}=\{(\tilde{X}_i,\tilde{Y}_i)\}_{i=1}^m$, i.e
  12 $$\begin{aligned} {\mathscr {J}}_{\alpha }:=\{j\in \{1,\dots ,N\} : \text{S}(\textbf{C}(\overline{P}_{\theta _j}(\tilde{\textbf{X}}),\tilde{\textbf{Y}}))) > \alpha \}. \end{aligned}$$
3. We define the ensemble prediction as the median value of all the selected predictions. Given a new sample *X*, we have
  13 $$\begin{aligned} \hat{Y}^{\theta }=m(\{\overline{P}_{\theta _j}(X): j\in {\mathscr {J}}_{\alpha }\}). \end{aligned}$$
  where *m* indicates the median function. In the case where the number of zeros is equal to the number of ones, we assume $\hat{Y}^{\theta }=1$.

In the second step of the above scheme, the parameter $\alpha$ in Eq. (12) has to be given. Differently from^41^, where the above procedure was introduced and $\alpha$ was arbitrarily chosen, we propose to compute it as follows.

(i) For each $\gamma \in [\gamma _0,\gamma _1)$ with $0<\gamma _0<\gamma _1<1$, consider the epochs for which the skill score $\text{S}$ computed on the validation set is higher than a given fraction $\gamma$ of the maximum possible score $\text{S}$ on the validation set by varying epochs
  14 $$\begin{aligned} {\mathscr {J}}_{\gamma }:=\{j\in \{1,\dots ,N\} : \text{S}(\textbf{C}(\overline{P}_{\theta _j}(\tilde{\textbf{X}}),\tilde{\textbf{Y}})) > \gamma \max _{l\in \{1, \dots ,N\}} \{ \text{S}(\textbf{C}(\overline{P}_{\theta _l}(\tilde{\textbf{X}}),\tilde{\textbf{Y}}))\} \}. \end{aligned}$$
  and compute the corresponding ensemble prediction on the validation set
  15 $$\begin{aligned} \hat{\textbf{Y}}^{\theta }_{\gamma }=m(\{\overline{P}_{\theta _j}(\tilde{\textbf{X}}): j\in {\mathscr {J}}_{\gamma }\}). \end{aligned}$$
(ii) Select the optimal parameter $\overline{\gamma }$ as the one which maximizes the skill score $\text{S}$ computed on the validation set
  16 $$\begin{aligned} \overline{\gamma }:=\arg \max _{\gamma \in [\gamma _0,\gamma _1)} \text{S}(\textbf{C}(\hat{\textbf{Y}}^{\theta }_{\gamma },\tilde{\textbf{Y}})) \end{aligned}$$
  and define the level $\alpha$ as follows
  17 $$\begin{aligned} \alpha :=\overline{\gamma }\max _{j\in \{1, \dots ,N\}}\{\text{S}(\textbf{C}(\overline{P}_{\theta _j}(\tilde{\textbf{X}}),\tilde{\textbf{Y}}))\}. \end{aligned}$$

As a result of this procedure, the estimated value of $\alpha$ only depends on the validation set.

**Figure 4:**
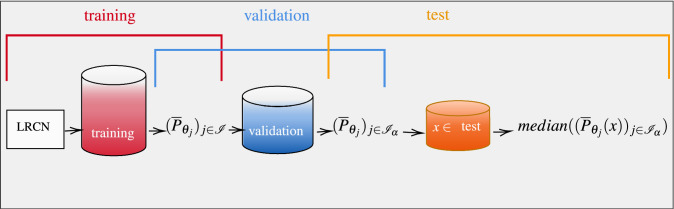
Pipeline diagram for the ensemble method. The first step consists in training the LRCN model (the architecture is shown in Fig. 2) over a fixed number of epochs and computing the classification thresholds defined in (10): the outputs of the training process are the estimators $(\overline{P}_{\theta _j})_{j\in {\mathscr {I}}}$ (see (11)) where ${\mathscr {I}}$ is the set of epoch indexes. The second step consists in validating the estimators $(\overline{P}_{\theta _j})_{j\in {\mathscr {I}}}$ by selecting the ones which provide predictions on the validation set with scores over a level $\alpha$, which is determined through the procedure described in Eqs. (14)–(17). The final step consists in testing the method on a new input: the prediction of the ensemble method is given by computing the median of the estimators $(\overline{P}_{\theta _j})_{j\in {\mathscr {I}}}$ applied on a new input *x*.

We show the pipeline diagram explaining the ensemble method in Fig. 4.

In order to ensure statistical robustness of the entire ensemble procedure, we repeat it *M* times by randomizing the initial values of the weights, i.e. by training the deep neural network *M* times and we take the best ensemble prediction on the validation set. The best prediction is in the sense of the highest preferred skill score $\text{S}$. Therefore, by denoting with $\theta ^{(k)}$ the weights of the trained deep neural network at the *k*-th random initialization, we define the optimal weights as

18 $$\begin{aligned} \overline{\theta }:=\arg \max _{(\theta ^{(k)})_{k=1,\dots ,M}} \text{S}'(\textbf{C}(\hat{\textbf{Y}}^{\theta ^{(k)}}_{\overline{\gamma }},\tilde{\textbf{Y}})), \end{aligned}$$

where $\hat{\textbf{Y}}^{\theta ^{(k)}}_{\overline{\gamma }}$ is the ensemble prediction on the validation set obtained at the *k*-th random initialization of the training process.

In the following we show the performance of the ensemble deep learning technique when the LRCN network is used for the problem of forecasting extreme rainfall events in Liguria.

## Experimental results

In order to assess the prediction reliability of our deep NN model, we considered a historical dataset of CAPPI composite reflectivity videos recorded by the Italian weather radar network in the time window ranging from 2018/07/09 at 21:30 UTC to 2019/12/31 at 12:00 UTC, each video being 90 minutes long. For the training phase, we considered the time range from 2018/07/09 at 21:30 UTC to 2019/07/16 at 10:30 UTC and label the videos with binary labels concerning the concurrent occurrence of an over-threshold rainfall event from MCM data and lightning strikes in its surroundings, as explained in “Constant altitude plan position indicator reflectivity data in Liguria” section. The training set contains 7128 samples overall, with 105 samples labeled with 1, i.e. corresponding to extreme events according to the definition given in “Constant altitude plan position indicator reflectivity data in Liguria” section. For the validation step, we considered the videos in the time range from 2019/07/19 at 14:30 UTC to 2019/09/30 at 12:30 UTC (the validation set is made of 1296 videos overall, with 48 videos labeled with 1). Eventually, the test set is made of the CAPPI videos in the time range between 2019/10/03 at 15:00 UTC and 2019/12/31 at 12:00 UTC (the test contains 1899 videos, and 33 of them are labeled with 1). The model is trained over $N=100$ epochs using the Adam Optimizer^52^ with learning rate equal to 0.001 and mini-batch size equal to 72. The class balanced cross-entropy defined in (2) is used as the loss function in the training phase, where the weights $\beta _0$ and $\beta _1$ are defined as the inverse of the number of samples labeled with 0 and with 1 in each mini-batch, respectively.

**Figure 5:**
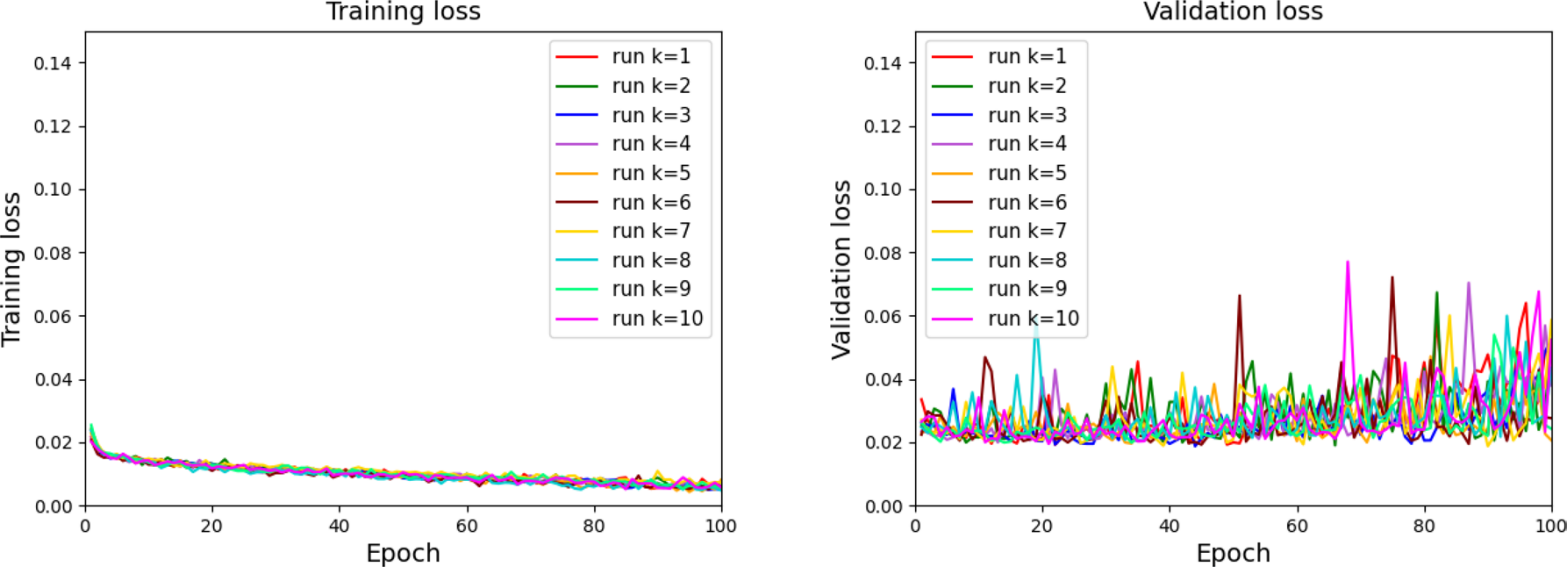
Learning curves showing the behaviour of the training (left panel) and validation (right panel) loss along epochs for the ten runs.

As explained in “Ensemble deep learning” section, the statistical significance of the results is guaranteed by running the network $M=10$ times, each time with a different random initialization of the LRCN weights. We report in Fig. 5 the training and validation loss per epoch for the 10 runs. We noticed that the validating loss curves have more fluctuations for some runs especially after 60 epochs: this is most probably due to the fact that the training and validation sets have different percentages of samples labeled with 1 for the chronological splitting. Finally, we applied the ensemble strategy as described in “During the iterative optimization proc” Section, using the TSS and wTSS for choosing the epochs with best performance. For sake of clarity, for now on the two ensemble strategies will be named as TSS-ensemble and wTSS-ensemble, respectively.

**Table 1:** Results on the test set obtained by using the TSS-ensemble and wTSS-ensemble strategies.

Strategy	Confusion matrix		TSS	CSI	wFP	wFN	wTSS	wCSI
wTSS	TN = $1725.40_{(\pm 21.98 )}$	FP = $140.60_{(\pm 21.98 )}$	$0.78_{(\pm 0.04 )}$	$0.17_{(\pm 0.02 )}$	$243.88_{(\pm 41.34 )}$	$6.79_{(\pm 1.64 )}$	$0.68_{(\pm 0.04 )}$	$0.10_{(\pm 0.02 )}$
	FN = $4.70_{(\pm 1.25 )}$	TP = $28.30_{(\pm 1.25 )}$						
TSS	TN = $1727.60_{(\pm 32.42 )}$	FP =$138.40_{(\pm 32.42 )}$	$0.77_{(\pm 0.05 )}$	$0.17_{(\pm 0.03 )}$	$240.99_{(\pm 60.57 )}$	$7.24_{(\pm 2.60 )}$	$0.67_{(\pm 0.06 )}$	$0.10_{(\pm 0.02 )}$
	FN = $5.10_{(\pm 1.85 )}$	TP = $27.90_{(\pm 1.85 )}$						

The entries are the average values of the scores over 10 runs of the network for 10 random initializations of the weights. The standard deviations are also included.

**Figure 6:**
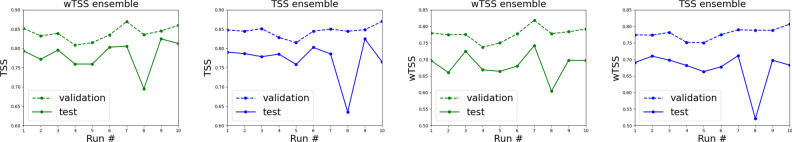
From right to left: TSS values on the validation set (dashed lines) and test set (continuous lines) obtained on each run by applying the wTSS-ensemble strategy (first panel) and the TSS-ensemble strategy (second panel); wTSS values on the validation set (dashed lines) and test set (continuous lines) obtained on each run by applying the wTSS-ensemble strategy (third panel) and the TSS-ensemble strategy (fourth panel).

**Figure 7:**
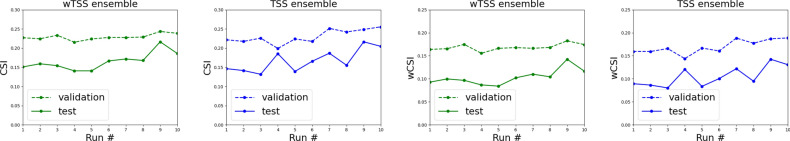
From left to right: CSI values on the validation set (dashed lines) and test set (continuous lines) obtained on each run by applying the wTSS-ensemble strategy (first panel) and the TSS-ensemble strategy (second panel); wCSI values on the validation set (dashed lines) and test set (continuous lines) obtained on each run by applying the wTSS-ensemble strategy (third panel) and the TSS-ensemble strategy (fourth panel).

**Table 2:** Results on the test set obtained by using the wTSS-ensemble strategy when the run is selected with respect to the best TSS or wTSS ($k=7$ run), the wTSS-ensemble strategy when the run is selected with respect to the best CSI or wCSI ($k=9$ run) and the TSS-ensemble strategy when the run is selected with respect to the best TSS or wTSS or CSI or wCSI ($k=10$ run).

Score	Strategy					
	wTSS ensemble				TSS ensemble	
	$\text{S}'=$TSS/wTSS (run $k=7$)		$\text{S}'=$CSI/wCSI (run $k=9$)		$\text{S}'=$TSS/wTSS/CSI/wCSI (run $k=10$)	
Confusion matrix	TN = 1730	FP = 136	TN = 1765	FP = 101	TN = **1767**	FP = **99**
	FN = **4**	TP = **29**	FN = **4**	TP = **29**	FN = 6	TP = 27
TSS	0.8059		${\textbf {0.8247}}$		0.7651	
CSI	0.1716		${\textbf {0.2164}}$		0.2045	
wFN	${\textbf {4.75}}$		8		8	
wFP	229.83		${\textbf {166.58}}$		171.67	
wTSS	${\textbf {0.742}}$		0.6975		0.6829	
wCSI	0.11		${\textbf {0.1425}}$		0.1306	

In bold the best results are highlighted.

These two strategies have been applied to the test set, and the results are illustrated in Table 1, where we reported the average values and the corresponding standard deviations for the entries of the quality-based and value-weighted confusion matrices, and for the TSS, CSI, wTSS, and wCSI. The table shows that the score values are all rather similar, although the averaged TSS and wTSS values are slightly higher when the wTSS-ensemble strategy is adopted.

Since, according to the ensemble strategy, the prediction for a specific test set is made by using the weights corresponding to the best run in the validation set, in Fig. 6 we show the behavior of TSS and wTSS for the TSS-ensemble and wTSS-ensemble strategies, in the case of 10 runs of the network corresponding to 10 random initializations of the weights.

**Figure 8:**
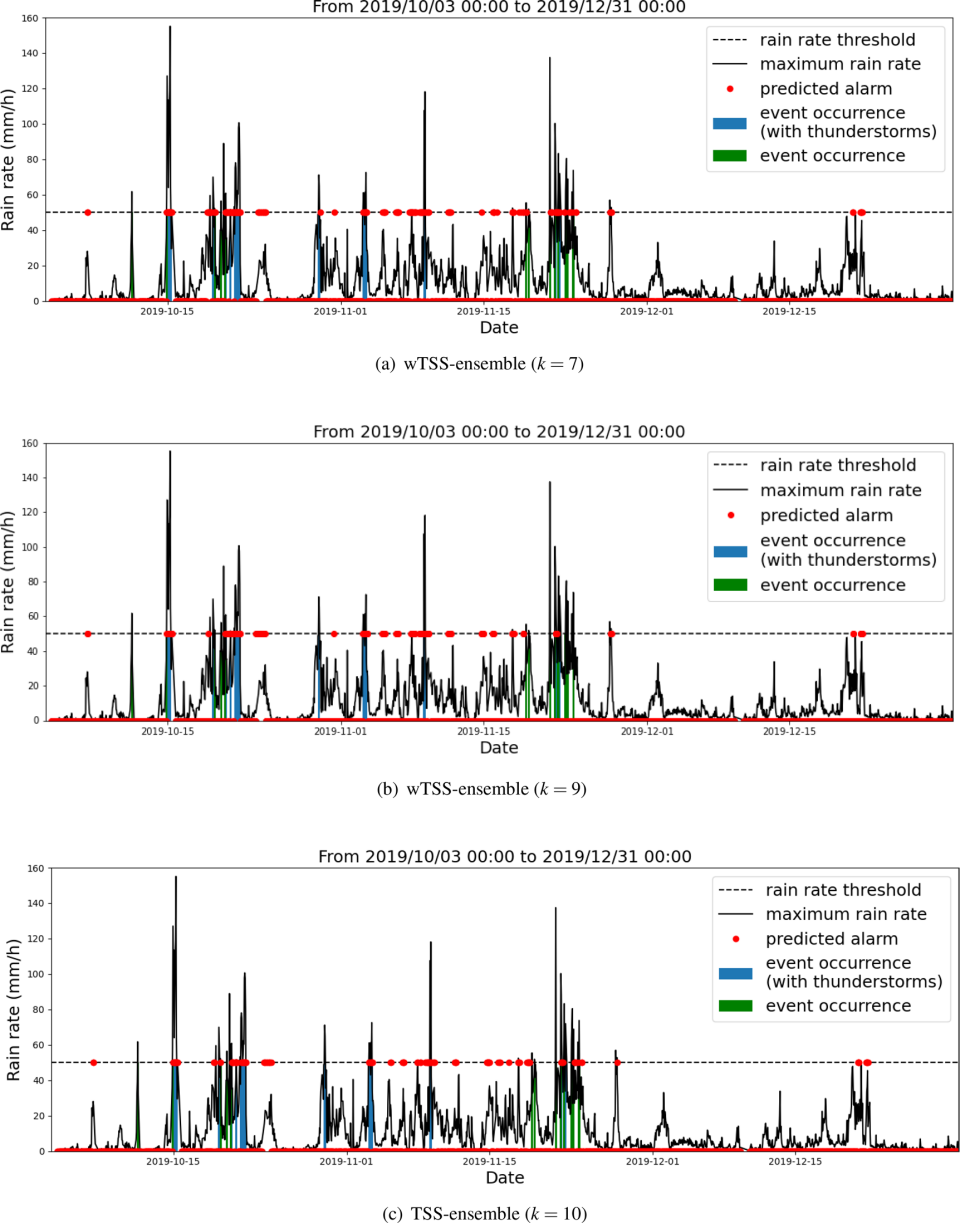
Predictions for the testing period obtained by applying the wTSS-ensemble strategy at $k=7$ run (top panel), the wTSS-ensemble strategy at $k=9$ run (central panel) and the TSS-ensemble strategy at $k=10$ run (bottom panel).

The results in Fig. 6 imply that, in the case of the wTSS-ensemble strategy, the best score values in validation correspond to the best score values in the test phase. Figure 7 illustrates the same analysis in the case when the scores used for assessing the prediction performance are CSI and wCSI and shows that, also in this case, the wTSS-ensemble strategy should be preferred. We pointed out that the gap between validation and test scores is most probably due to the heterogeneity of the data used in training, validation and test sets: the test set represents mainly the autumnal period whereas the validation comprises mainly data of the summer period. We think that a better practice could be using data of the autumnal period of many past years for training and validating the network in order to have a better prediction on the next autumn.

Table 2 contains the values of confusion-matrix entries and scores obtained by using the weights associated to the best runs of the network selected during the validation phase by means of the TSS-ensemble and wTSS-ensemble strategies. Please consider that in the case of the TSS-ensemble strategy, the best run is always the $k=10$ one.

**Figure 9:**
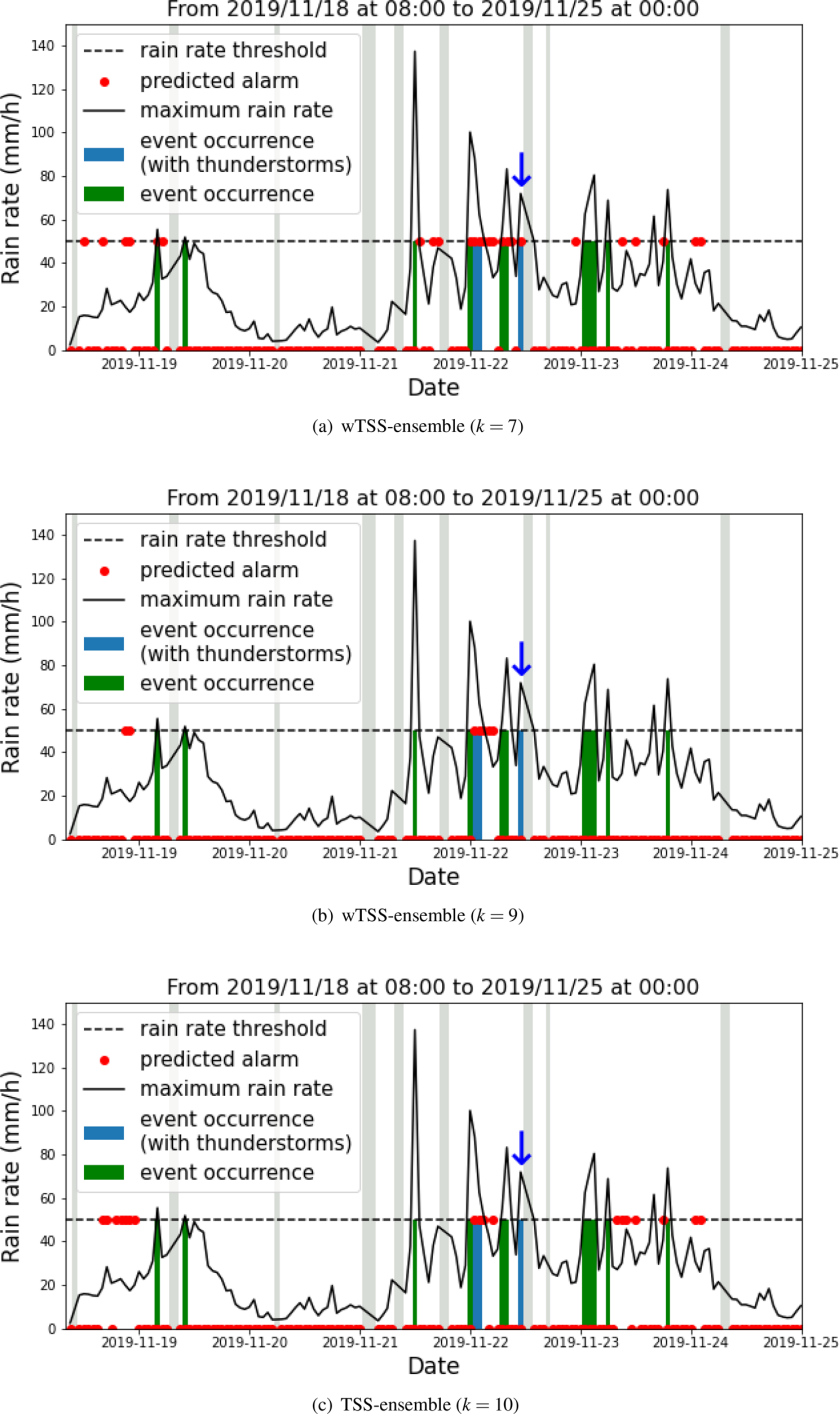
Predictions valid from 2019/11/18 at 08:00 UTC to 2019/11/25 at 00:00 UTC obtained by applying the wTSS-ensemble strategy at $k=7$ run (top panel), the wTSS-ensemble strategy at $k=9$ run (central panel) and the TSS-ensemble strategy at $k=10$ run (bottom panel). The grey boxes correspond to times when the input data are missing.

In order to show how the use of value-weighted scores performs in action, in Fig. 8 we enrolled over time the predictions corresponding to the test set, when the wTSS-ensemble and TSS-ensemble strategies are adopted and when wTSS, TSS, wCSI and CSI are used for selecting the best run (we point out again that using wTSS and TSS for the wTSS-ensemble strategy always leads to $k=7$ and that using wCSI and CSI for the same ensemble strategy always leads to $k=9$).

We remind that the labeling procedure depends on the rain rate and on the presence of lighting, as described in “Constant altitude plan position indicator reflectivity data in Liguria” Section. The blue bars represent the events labeled with 1, i.e. events which satisfy the condition on both the rain rate and the presence of lighting, whereas the green bars are events that satisfy only the condition on the rain rate.

We first point out that when the wTSS-strategy is used and $k=7$ is selected, the prediction tends to systematically anticipate the events characterized by high rain rate. Further, for sake of clarity, Fig. 9 contains a zoom around the November 22 2019 time point, when a dramatic flood caused significant damage in many areas of Liguria. This zoom shows that the wTSS-ensemble strategy for $k=7$ is able to correctly predict the thunderstorms occurring in the time interval from 00:00 to 02:00 UTC on November 22 2019 and to anticipate the other catastrophic thunderstorm occurring between 10:00 and 11:00 UTC (this last thunderstorm is marked with a blue arrow in all panels of Fig. 9). No anticipated alarm is sounded by the other two predictions.

## Conclusions and future work

The realization of warning machines able to sound binary alarms along time is an intriguing issue in many areas of forecasting^53–56^. The present paper shows for the first time that a deep CNN exploiting radar videos as input can be used as a warning machine for predicting severe thunderstorms (in fact, previous CNNs in this field have been used to synthesize simulated radar images at time points successive to the last one in the input time series). It is worth noticing that the aim here is not the prediction of the exact location and intensity of a heavy rain event, but rather the probable occurrence of a severe thunderstorm over a reference area in the next hour.

The crucial point in our approach relies on the kind of evaluation metrics adopted. In fact, the TSS can be considered a good measure of performance in forecasting, since it is insensitive to the class-imbalance ratio. However, such a skill score, as all the ones computed on a classical quality-based confusion matrix, does not account for the temporal distribution of alarms. Therefore, we propose to focus on value-weighted skill scores, as the wTSS, which account for the distribution of the predictions over time while promoting predictions well in advance. We focused on the problem of forecasting extreme rainfall events on the Liguria region, and we showed that the performance of our ensemble technique in the case when wTSS is optimized, is significantly better than the performance when the model is trained to optimize a standard quality-based score.

Next in line in our work will be the application of a class of score-driven loss functions^57^, whose minimization in the training phase allows the automatic maximization of the corresponding skill scores. Possible future studies of this work concern (1) the investigation of other ensemble techniques as^58,59^, (2) the use of feature selection methods which allow individuating the most relevant subset of features extracted by CNN models as in^60^, (3) the use of dynamic graph modeling approaches to learn spatial-temporal representations in radar reflectivity videos^61^. Further, deep hashing methods^62^ could be used to exploit more information for the prediction, like the density and type of lightning (such as cloud-to-cloud and cloud-to-ground strikes).

## Data Availability

The data that support the findings of this study are available from the Italian Civil Protection Department (radar data) and the Italian Military Aeronautic (lightnings data) but restrictions apply to the availability of these data, which were used under license for the current study, and so are not publicly available. Data are however available from the authors upon reasonable request and with permission of the Italian Civil Protection Department (radar data) and the Italian Military Aeronautic (lightnings data). However, the radar data can be downloaded from https://mappe.protezionecivile.gov.it/it/mappe-rischi/piattaforma-radar and we put at disposal the code of the deep neural network and the ensemble procedure in the github repository https://github.com/SabrinaGuastavino/Ensemble-deep-learning.
